# Supplementary material for: Tead1a Initiates Transcriptional Priming Through the TEAD1a/YAP‐Notch1‐Spi1/Cebpα Axis to Promote Neutrophil Fate
Source: Adv Sci (Weinh). 2025 Aug 28;12(41):e05441. doi: 10.1002/advs.202505441 (PMC12591127; doi:10.1002/advs.202505441)
Supplement: Supplementary file 4 — Supplementary Dataset [file ADVS-12-e05441-s004.zip › 3-4.zip!/3-4/02-4.cluster/cluster9/Enrich/ego_CC.pdf]

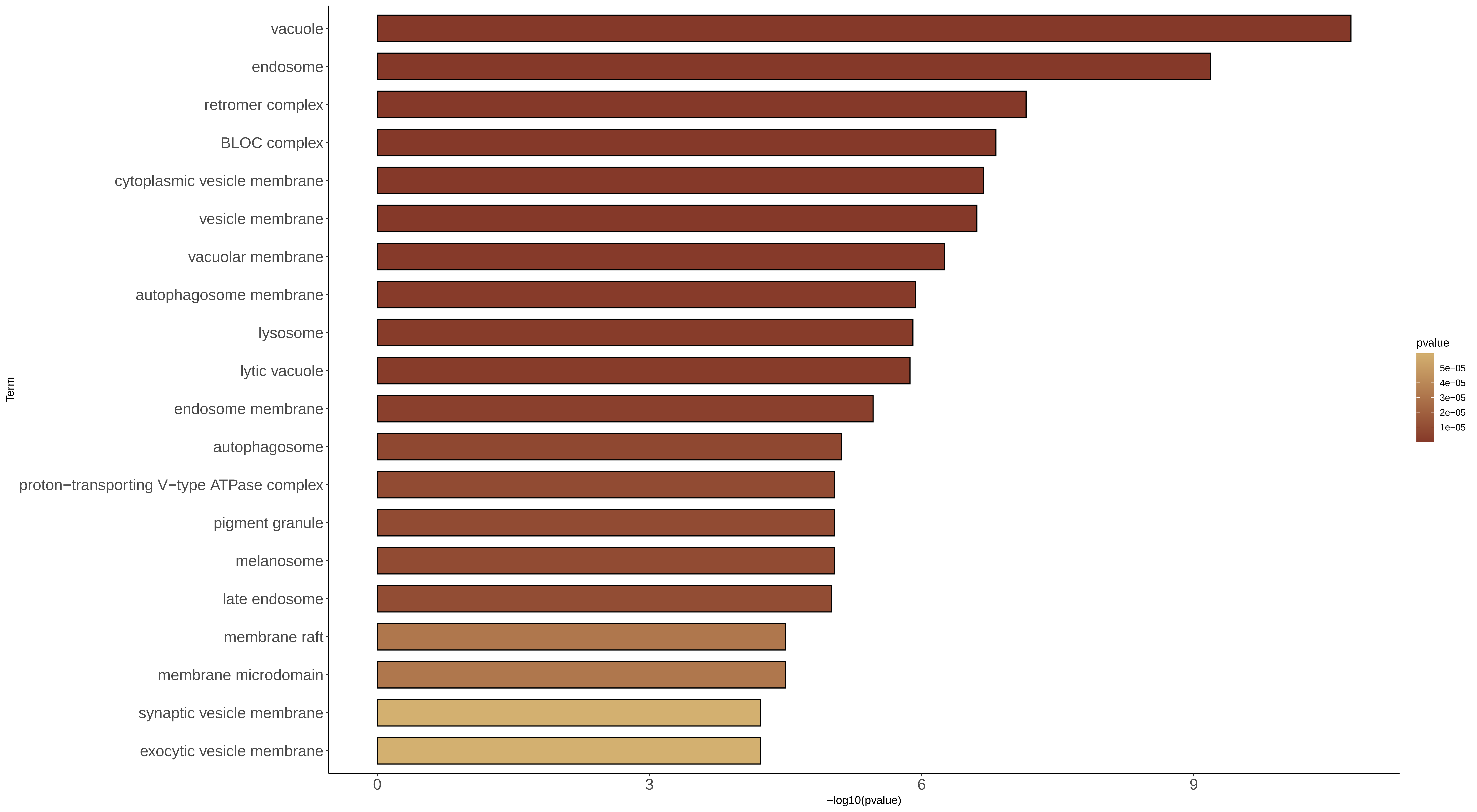
vacuole
endosome
retromer complex
BLOC complex
cytoplasmic vesicle membrane
vesicle membrane
vacuolar membrane
autophagosome membrane
lysosome
lytic vacuole
endosome membrane
autophagosome
proton−transporting V−type ATPase complex
pigment granule
melanosome
late endosome
membrane raft
membrane microdomain
synaptic vesicle membrane
exocytic vesicle membrane
Term
0
3
6
9
−log10(pvalue)
pvalue
5e−05
4e−05
3e−05
2e−05
1e−05
